# The Impact of Phenylalanine Levels on Cognitive Outcomes in Adults With Phenylketonuria: Effects Across Tasks and Developmental Stages

**DOI:** 10.1037/neu0000336

**Published:** 2017-03

**Authors:** Cristina Romani, Liana Palermo, Anita MacDonald, Ellie Limback, S. Kate Hall, Tarekegn Geberhiwot

**Affiliations:** 1 School of Life and Health Sciences, Aston University; 2 School of Life and Health Sciences, Aston University; Queen Elizabeth Hospital, Birmingham, England; and Department of Medical and Surgical Sciences, Magna Graecia University of Catanzaro; 3 Birmingham Children’s Hospital, Birmingham, England; 4 School of Life and Health Sciences, Aston University; 5 UK Newborn Screening Laboratories Network, Oxford, England; 6 Queen Elizabeth Hospital, Birmingham, England

**Keywords:** PKU, cognitive skills, phenylalanine fluctuations, development, memory and learning

## Abstract

***Objective:*** Phenylketonuria (PKU) is due to an inability to metabolize the amino acid phenylalanine (Phe), leading to its accumulation in the brain. Phe levels can be controlled following a protein-free diet, but cognitive impairments are still present. A number of questions remain to be answered related to which type of metabolic control is important, the age when it is important, the cognitive functions which are most affected and, the best tests to use to monitor cognitive health. ***Method:*** We investigated the impact of metabolic control at different ages on cognitive performance in 37 early treated adults with PKU. ***Results:*** (a) Phe variation was as associated to performance as average Phe showing that stable dietary control is as important as strict control; (b) For some tasks, current and adult Phe were stronger predictors of performance than childhood or adolescent Phe, showing the importance of a strict diet even in adulthood; and (c) The relationship between performance and Phe levels varied depending on time and cognitive domain. For some functions (sustained attention, visuomotor coordination), Phe at the time of testing was the best predictor. While for other functions (visual attention, executive functions) there was a diminishing or stable relationship across time. ***Conclusion:*** Results show the importance of selecting the right tasks to monitor outcomes across ages, but also that the impact of bio-chemical disruptions is different for different functions, at different ages. We show how inherited metabolic diseases offer us a unique vantage point to inform our understanding of brain development and functioning.

Phenylketonuria (PKU) is an inherited metabolic disease where an enzyme deficiency results in an inability/reduced ability to metabolize the amino acid phenylalanine (Phe) into tyrosine. This deficiency results in an increased concentration of Phe in the blood and in the brain which, in turn, causes brain abnormality and cognitive impairment (Blau, van Spronsen, & Levy, 2010). The toxic effects of Phe can be minimized by a low Phe diet commenced at the time of diagnosis, in infancy. However, the diet is very restricted (Phe is present in most protein foods) and, thus, unsociable and expensive for health services (protein supplements have to be used), which results in variable dietary adherence, especially after adolescence when the toxic effects of Phe are unclear. This variable adherence is possibly one of the main reasons why cognitive outcomes are extremely variable in adults with PKU (AwPKU) with some individuals performing extremely well across cognitive domains and others showing significant impairments (see Palermo et al., 2017). However, robust evidence of the importance of treatment after adolescence is lacking (see later for a review) and this has led to inconsistent recommendations. Since treatment was introduced, the recommendation to maintain a strict diet has changed from discontinuation at 6 years old (see Berry et al., 2013) to treatment for life both in the United States (Vockley et al., 2014) and Europe (MacDonald personal communication, forthcoming at http://www.espku.org/who-we-are/european-guidelines), but how strongly diet for life is recommended across clinical centers remains inconsistent. We need strong evidence of the impact of high-Phe level on cognition to support clinical recommendations.

A number of studies have highlighted impairments in speed of processing and in complex executive functions involving planning, switching, and monitoring (see Moyle, Fox, Arthur, Bynevelt, & Burnett, 2007; Christ, Huijbregts, de Sonneville, & White, 2010). We have also documented these impairments in a group of AwPKU, while, at the same time, reporting normal performance in other domains (spelling, naming, verbal and visuospatial memory and learning; see Palermo et al., 2017). However, although it is clear that levels of Phe impact cognition in children (Waisbren et al., 2007), their impact in adults is less clear. We have found only 14 studies that have assessed relationships between Phe levels and cognitive functions in AwPKU, with mixed results (Bik-Multanowski, Pietrzyk, & Mozrzymas, 2011; Brumm et al., 2004; Channon, German, Cassina, & Lee, 2004; Channon, Goodman, Zlotowitz, Mockler, & Lee, 2007; Channon, Mockler, & Lee, 2005; Moyle, Fox, Bynevelt, Arthur, & Burnett, 2007; Nardecchia et al., 2015; Pietz et al., 1998; Ris, Williams, Hunt, Berry, & Leslie, 1994; Schmidt et al., 1994; Smith, Klim, Mallozzi, & Hanley, 1996; Sundermann et al., 2011; ten Hoedt et al., 2011; Weglage et al., 2013).

## Literature Review

Relationships with cognitive functions have been assessed by contrasting performance when Phe levels are high or low or by treating Phe as a continuous variable and assessing correlations.

### Comparing Effects of High Versus Low Average Phe

Group of PKU participants have been subdivided using arbitrary cut-offs for average level of Phe at the time of testing, usually above versus below 1,000 μmol/L (Bik-Multanowski et al., 2011; Brumm et al., 2004; Smith et al., 1996); by comparing off-diet and on-diet individuals (Channon et al., 2007; Schmidt et al., 1994); or by contrasting performance in the same participants after Phe level has been manipulated through pharmacological intervention (Sundermann et al., 2011; ten Hoedt et al., 2011). Across these studies (*N* = 7), 64 comparisons were made with different cognitive measures, of which 25% yielded significant results in the expected direction with worse performance with higher Phe. Significant results, however, were found in different tasks and cognitive domains by different studies (e.g., complex executive functions such as the Wisconsin Card Sorting Test, Smith et al., 1996; working memory, Bik-Multanowski et al., 2011; Channon et al., 2007; inhibitory control, Bik-Multanowski et al., 2011; Channon et al., 2007; spoken language, Brumm et al., 2004; and sustained attention, Bik-Multanowski et al., 2011; Schmidt et al., 1994; ten Hoedt et al., 2011).

### Correlations Between Cognitive Performance and Phe Levels

Other studies (*N* = 8) have looked at associations between levels of Phe and cognitive performance through correlations. Most have looked at correlations with current Phe (Brumm et al., 2004; Channon et al., 2004, 2005, 2007; Moyle, Fox, Bynevelt, et al., 2007; Pietz et al., 1998; Ris et al., 1994; Smith et al., 1996) finding significant associations for a subset of tasks (18/87 = 21% across studies) and with these tasks tapping different domains depending on the study (i.e., complex executive functions: Moyle, Fox, Bynevelt, et al., 2007; Smith et al., 1996; Ris et al., 1994; working memory: Channon et al., 2004; spoken language: Brumm et al., 2004; verbal memory: Moyle, Fox, Bynevelt, et al., 2007, and visual memory: Moyle, Fox, Bynevelt, et al., 2007). Finally, a few studies (*N* = 5) have also looked at correlations between adult cognitive performance and historic levels of Phe during childhood and/or adolescence (Brumm et al., 2004; Channon et al., 2004, 2005, 2007; Weglage et al., 2013). Again, these studies have found significant correlations for a subset of tasks across different cognitive domains (17/57 = 29.8%). Comparing the size of correlations obtained with current versus historical Phe levels is not easy because different studies looked at Phe levels across different time bands. However, there are no clear differences supporting the idea that early metabolic control is particularly important in determining adult performance and yields stronger correlations.

In spite of variability, current results do show that cognitive performance in AwPKU is related to present and past levels of Phe. It is unlikely that the 20–30% of significant outcomes are obtained by chance. Which cognitive functions do or do not show a relationship with Phe and at which ages, however, remains unclear. Across the reviewed studies, the impact Phe levels on cognitive performance has been examined with as many as 87 different tasks across a variety of cognitive domains. This may seem an impressive number of tasks, but only a few tasks have been used by each study (with the exception of Brumm et al., 2004, with a relatively small number of participants: *N* = 20). This makes it difficult to assess whether associations vary depending on cognitive domain. We need studies that assess the same functions in the same participants to properly allow comparisons.

## Predictions

One can entertain different hypotheses of how poor metabolic control may impact on different functions, at different ages. In adults, there is some indication that executive functions are more affected by current Phe levels than measures related to speed of processing (Bik-Multanowski et al., 2011; Channon et al., 2005; Moyle, Fox, Bynevelt, et al., 2007). A review by Albrecht, Garbade, and Burgard (2009) showed a diminishing association between Phe levels and speed of processing from childhood to adulthood. Speed of processing relies on fast connections between brain areas. It is possible that the toxic effect of Phe on myelin (see Möller et al., 2003; Moyle, Fox, Arthur, et al., 2007) causes structural damage, which is relative insensitive to Phe differences in adulthood. On the contrary, executive functions and sustained attention may be more sensitive to a reduction in dopamine levels caused by high Phe levels (PKU reduces the amount of endogenous tyrosine and tyrosine is a precursor of dopamine; Burlina et al., 2000; McKean, 1972; Puglisi-Allegra et al., 2000). There may also be interactions depending on the maturation time of different functions. For example, functions that mature earlier—such as visuospatial functions—may show a stronger effect of early metabolic control compared to functions maturing later—such as some executive functions (for differences in maturation rates see, Best & Miller, 2010; Gogtay et al., 2004; Klingberg, Vaidya, Gabrieli, Moseley, & Hedehus, 1999)—which may show stronger association with adolescent Phe. All of these hypotheses need to be verified through studies where these functions are examined in the same participants and put in relation with metabolic control at different ages.

How to identify the best tasks sensitive to high-level of Phe is also unclear. One may assume that the functions that are most affected by high blood Phe are those showing larger group impairments (see van Spronsen, Huijbregts, Bosch, & Leuzzi, 2011, for such an argument). This, however, may be unwarranted. Group impairments depend on a function being systematically impaired across individuals. Correlations, instead, depend crucially on variability. If all individuals in the group are impaired irrespective of current levels of Phe, no correlation or modest correlations will be seen. Correlations, instead, may be stronger when performance in the group is heterogeneous because only the individuals with the highest Phe are impaired, whereas the others perform normally. This predicts correlations even for functions where, as a group, AwPKU do not show a significant impairment. This is particularly important for AwPKU who have largely maintained good metabolic control throughout their life. In these groups, impairments will be subtler and restricted to some cognitive areas and/or to individuals with worse control.

## Plan of Study

Our study wants to assess the predictions outlined above by testing a group of 37 AwPKU with relatively good metabolic control (with averages <500 μmol/L in childhood; <850 μmol/L in adolescence and adulthood). Cognitive performance and variability for the same group of PKU participants was described in Palermo et al. (2017). Metabolic control at different ages was available through extensive historic records, allowing us to compare effects in childhood (>10 years), adolescence (11–17 years), and adulthood, as well as at time of testing, on cognitive performance in adulthood. We will assess the relationships both by dividing our participants into groups defined by metabolic control (better and worse) and by carrying out correlations with continuous measures of metabolic controls.

As in previous studies, we have measured mean Phe levels by averaging yearly median levels within a time period. In addition, Phe fluctuations were measured by averaging yearly standard deviations. Recent evidence from children studies suggest that variability in Phe levels is equally important (for effect on IQ, see Burgard et al., 1996; Hood, Grange, Christ, Steiner, & White, 2014; Vilaseca et al., 2010; for effect on executive functions, see Arnold et al., 1998; Hood et al., 2014; for a review see Cleary et al., 2013).

We have assessed cognitive performance across a number of cognitive domains including visuospatial attention, visuomotor coordination, complex executive functions, inhibitory control, short-term memory (STM), sustained attention, orthographic processing, spoken language, verbal memory and learning, and visual memory and learning. For tasks of visuospatial attention, picture naming and word reading both accuracy and speed measures were taken. We assessed correlations with measures of orthographic processing (usually not reported in the literature) because they are highly affected by education and socioeconomic status. Correlations between Phe and cognitive measures may be spuriously mediated by socioeconomic variables, which may be associated with both better cognitive performance and better dietary control. If correlations with orthographic proficiency are not present, this will strengthen our confidence that cognitive performance is truly affected by Phe levels.

Results will provide helpful information to (a) justify recommendations to maintain treatment for life; (b) identify best measures to monitor cognitive outcomes depending on dietary control and developmental age; and (c) reach a better understanding of the neurophysiological basis of this disease.

## Method

### Participants and Procedure

Thirty-seven early treated participants with classical PKU were recruited from a pool of 88 individuals followed by the Department of Inherited Metabolic Disorders at Queen Elisabeth Hospital, in Birmingham who had been continuously treated with a low-Phe diet since diagnosis in infancy and were still contactable. All individuals who responded to the invitation were tested (*N* = 37). At the time of testing seven participants were on an unrestricted diet and 30 on a low-protein diet. The PKU participants were compared to a group of 30 healthy control participants recruited for the purpose of this study. The control group was matched to the PKU group for age, gender, and educational status (see Palermo et al., 2017, for further details). Data on historical Phe levels were obtained from the PKU database at the Clinical Chemistry Department at Birmingham Children Hospital. Diagnosis was through newborn screening conducted at 5–7 days after birth. Demographic and Phe levels across the life span are reported in Table 1.[Table-anchor tbl1]

Participants were tested in a quiet room in two separate testing sessions, each lasting about 3 hours. The average interval between testing session was 37 days (*SD* = 43). Blood Phe concentrations were measured prior to each testing session; values were then averaged to determine current Phe level. The research was approved by the NHS and Aston University Ethics committees. All participants gave voluntary informed consent to take part. All efforts were made to administer all tasks to all participants, but some data points were unavailable due to some PKU participants (*n* = 6) failing to attend a second appointment and some technical errors resulting in some missing data for two participants.

Our sample was divided into two subgroups on the basis of average Phe level from 17 years of age to present <650 versus >950 μmol/L. These valued were selected because they allowed us to divide our sample in two equal groups (*N* = 14 and 14), while, at the same time, including in the group with good control individuals with values close to current guidelines.

Phe levels in adulthood are strongly correlated with levels at the time of testing (current Phe) and with adolescent measures, but less with childhood measures (see Table 2).[Table-anchor tbl2]

### Tasks

As common practice, we used several tasks within each cognitive domain to reduce nondomain task-related variance. Our tasks were classified according to their primary assessment focus, but a degree of arbitrariness was unavoidable. For example, tasks like the digit symbol and the similarity subtest of the WASI are complex tasks that could have been classified among complex executive functions rather than within visuomotor coordination and language processing, respectively, as in this study.

IQ was measured using the Wechsler Abbreviated Scale of Intelligence (WASI; Wechsler, 1999), which includes the following subtests: Vocabulary, Block Design, Similarities, and Matrix Reasoning. In addition, participants were given an extensive neuropsychological battery including 28 different tasks providing 36 cognitive measures. Our results and measures tapped eight different cognitive domains.

#### Visuospatial attention

This included measures from six tasks: (a) simple detection—press a response button as soon as a ladybug appeared on the screen; (b) choice reaction time—press either a left or right response key consistent with the direction of an arrow centrally presented; (c) detection with distractors—press a button whenever a ladybug appeared on the screen alone or with a green bug; (d) opposite detection with distractors—press a button whenever a green bug appeared on the screen alone or with a ladybug; (e) feature search—detect a target among distractors not sharing features by pressing a “yes” or “no” button (e.g., a red ladybug among green bugs); and (f) conjoined search—Detect a target among distractors sharing features (e.g., red ladybug among red bugs and green ladybugs). Both reaction time (RT) and accuracy measures (error rates) were taken.

#### Visual memory and learning

This included measures from two tasks: (a) delayed matching to sample—recognize a previously seen pattern among distractors and b) paired associates visual learning—learn to associate objects with locations.

#### Visuomotor coordination

This included measures from two tasks: (a) grooved pegboard test—put pegs into the holes of a board using only one hand as quickly as possible and (b) digit symbol task—fill as many boxes as possible with symbols corresponding with numbers in 90 s.

#### Complex executive functions

This included measures from four tasks that, to be completed, involve a number of complex functions including planning, flexibility, and abstract thinking as well as to some more specific functions (working memory/STM, sustained attention, inhibitory control that we examined separately, see below; see also Miyake et al., 2000): (a) The Wisconsin Card Sorting Test-64 Card Version—discover the rules to match cards from a deck with four reference cards according to shape, number and color using feedback. Flexibility is required when the sorting rule is changed unknown to the participant and the new rule has to be discovered. Each incorrectly matched card was counted as an error. As a general score, we considered the total number of errors made by a participant (maximum number of errors = 64). (b) Difference in speed between Trail Making Test B-A (A involves connecting circles containing numbers in ascending order as quickly as possible; B also involves connecting circles in ascending order but alternating between number and letters). Only speed measures were taken because errors are rare. (c) Verbal (semantic) fluency—generate as many names of animals as possible in 1 min of time. This requires planning an efficient search through the lexicon. (d) The Tower of Hanoi puzzle—move a number of rings of different sizes across three pegs to form a tower on the last peg following specific constraints. Our score is based on the percentage of solved trials of different complexity (three, four, and five rings).

#### Inhibitory control

This included measures from two tasks: (a) Stroop interference—difference in RTs and errors between reporting the ink color of words where the color of the ink was incongruent with the meaning of the word (*red* written with yellow ink), or congruent (*red* written with red ink). (b) Semantic interference—differences in naming RTs and errors between the first and the last item in a series of semantically related pictures. A number of studies have demonstrated reliable differences due to a building up of interference from semantic competitors (see original study by Howard, Nickels, Coltheart, & Cole-Virtue, 2006). One hundred sixty-five pictures were presented one at time on a computer screen and participants were instructed to say the name as soon as possible. One hundred twenty pictures belonged to 24 different semantic categories (five items in each category) and the remaining 45 were fillers. The number of pictures between successive members of the same category varied from two to eight. RTs were recorded via a voice-key. Previous results have shown that both RTs and errors progressively increase with the ordinal position in a set of semantically related pictures (Howard et al., 2006; Oppenheim, Dell, & Schwartz, 2007). Differences in accuracy and RT between the first and last exemplar of a set were taken as an index of the difficulty in controlling lexical semantic interference.

#### STM/working memory

This included measures from three tasks: (a) digit span—repeat a sequence of digits spoken by the examiner, soon after presentation; (b) nonword repetition—repeat a sequence of nonwords spoken by the examiner, soon after presentation; and (c) the Corsi block tapping test—the examiner taps a sequence of blocks and the participant has to reproduce the sequence in the same order.

#### Sustained attention

Percentage correct from the rapid visual information processing task—in a continuous series of numbers, detect three target sequences of three digits by pressing the response key when the last number of the sequence appears on the screen. To minimize the need of STM the written target sequences were kept in view of the participants.

#### Orthographic language

This involved measures from five tasks: (a and b) word and nonword reading—read as fast as possible an English or a made up word and both RT and accuracy measures (error rates) were taken; (b) spelling—spell words/nonwords to dictation; (d) phoneme deletion—delete a sound from a word (e.g., powder; /d/ > power); (e) spoonerisms—exchange the initial sounds of two words to produce two different words (e.g., bad-sin > sad-bin). These last two tasks were included because performance shows strong correlations with orthographic skills (Landerl, Wimmer, & Frith, 1997; Romani, Tsouknida, & Olson, 2015).

#### Spoken language

This included measures from four tasks: (a) picture naming—name a picture as fast as possible; (b) color naming—name as fast as possible the ink color of three X’s or colored words; only the congruent condition is considered here, where the color of the ink matched the meaning of the word, for example, *red* written with red ink. Both RT and accuracy measures (error rates) were taken; (c) similarities from the WASI—describe how similar in meaning two words are; and (d) vocabulary from the WASI—define a word.

#### Verbal Memory and learning

This included measures from two tasks: The Rey Auditory Verbal Learning Test (learning, immediate recall and delayed recall of a list of 15 words; Schmidt, 1996) and paired associates verbal learning (learning the association between a made-up word and the picture of an object or animal). 9 pairings with 5 learning trials.

### Analyses

When different measures were available for a cognitive domain, we aggregated results by averaging z scores. Accuracy and speed measures were similarly averaged. Z scores were calculated in terms of standard deviations from the control group. Higher z scores always reflect worse performance. We report both average z scores and % of impaired cognitive scores out of all scores computed. This second summary measure is important because PKU participants may not show an overall level of performance very different from controls, but still show deficits in a significant number of tasks.

## Results

Overall group differences by cognitive domains are presented in Table 3. AwPKU were significantly impaired compared to controls in most cognitive domains with the exception of inhibitory control and memory and learning. The impairment in visuomotor coordination was particularly severe. For more details on group performance on individual tasks, see Palermo et al. (2017). Group impairments were evident both in terms of overall z score and percent of impaired scores.[Table-anchor tbl3]

### Differences Between Individuals With Good Versus Poor Control

Demographic and metabolic information for the two groups, their IQ, and measures of overall cognitive performance are reported in Table 4. The two groups do not differ in education, but the group with poorer control is older. The two groups already differ in metabolic control in childhood, but differences become stronger in adolescence and adulthood. Importantly, the two groups differ in cognitive performance. The group with higher Phe has a marginally lower IQ, a significantly higher overall z score, and a significantly higher percentage of impaired scores (>2 z scores from control group). Differences between the low-Phe group and the control group were modest but remained significant, IQ: *t*(1,42) = 1.98; *p* = .054; overall z scores: *t*(1,42) = −2.7; *p* < .01; % of impaired measures: *t*(1,42) = −2.5; *p* = .02.[Table-anchor tbl4]

**Figure 1 fig1:**
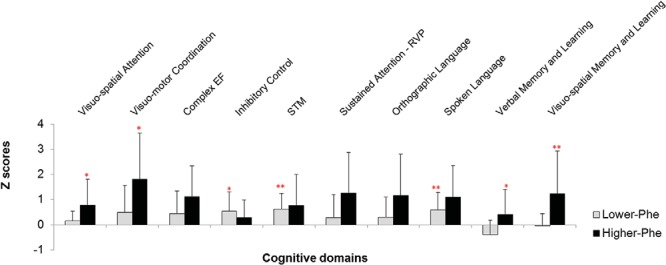
Comparison of cognitive abilities in lower- and higher-Phe phenylketonuria groups. Results are in z scores from the control group. Higher z scores always indicate worse performance. The asterisks on the lower-Phe group indicate a significant difference with controls; the asterisks on the higher-Phe group refer to a significant difference with the lower-Phe groups. RT = reaction time. * *p* < 0.05. ** *p* < 0.01. See the online article for the color version of this figure.

Results by cognitive domain are shown in Figure 1. In all domains, except inhibitory control, the lower-Phe group performed better than the higher-Phe group. Differences reached significance for visuospatial attention, *t*(1,26) = −2.1; *p* < .05; visuomotor coordination, *t*(1,26) = −2.3; *p* < .05; visuospatial memory and learning, *t*(1,26) = −2.7; *p* = .01; and verbal memory and learning, *t*(1,20) = −2.4; *p* < .05. All these differences remained significant when we covaried number of Phe specimen which differed between groups. Covarying age, differences between groups remained significant for visuomotor coordination, *t*(1,25) = 5.2; *p* = .03, and visuospatial memory and learning scores, *t*(1,25) = 4.2; *p* = .05, whereas differences in verbal memory and learning, *t*(1,19) = 3.9; *p* = .06, approached significance. Overall, the lower-Phe group performed very close to the controls even if significant differences remained across most domains.[Fig-anchor fig1]

### Correlations Between Metabolic and Cognitive Variables

**Figure 2 fig2:**
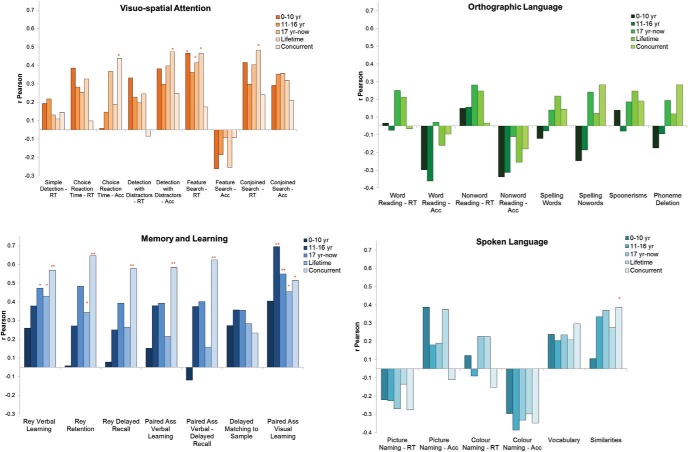

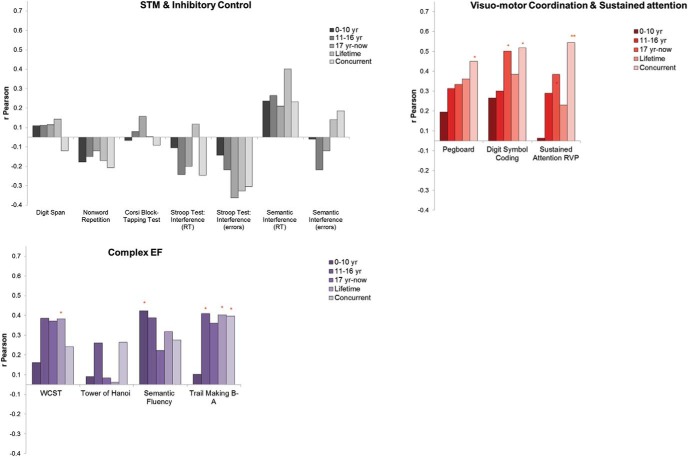
Correlations by cognitive domain and metabolic control as average Phe. EF = executive functions; STM = short-term memory; RVP = Rapid visual information processing. See the online article for the color version of this figure.

Table 5 reports Pearson’s R correlations between performance in different cognitive domains obtained in adulthood and continuous measures of metabolic control from different time bands, in terms of (a) blood Phe averages, (b) blood Phe fluctuations, and (c) an overall measure of metabolic control which combined average and fluctuations using within group z-scores. For ease of interpretation, for all tasks, we have reported as positive those correlations showing worse performance with higher the Phe (e.g., a correlation between high Phe and low IQ is reported as positive as well as a correlation between high Phe and slow RTs). Correlations for individual tasks are shown in Figure 2.[Table-anchor tbl5][Fig-anchor fig2]

#### Differences between domains

Not all correlations were significant (in Table 5 considering both correlations with Phe averages and Phe fluctuations: 43/99 = 43% are significant). It is important to note, however, that nonsignificant correlations concentrate on a few domains. There are no significant correlations with inhibitory control, STM, and orthographic language and correlations with spoken language are limited (no correlations with Phe average, but correlations with Phe fluctuation). In all other domains, the great majority of correlations were significant across different metabolic measures (Phe averages and Phe SDs), with measures taken at different times, and with good consistency across individual tasks. This makes it very unlikely that positive results were obtained by chance. For example, Figure 2 shows that the number of positive correlations for most cognitive domains is much higher than would be expected by chance when an equal number of associations should be positive and negative: for visuospatial attention: 40/45 (χ^2^ = 16.1; *p* < .001); memory and learning: 33/35 (χ^2^ = 17.2; *p* < .001); visuomotor coordination: 10/10 (χ^2^ = 6.7; *p* = .01); sustained attention: 5/5 (χ^2^ = 3.4; *p* = .06); complex executive functions: 20/20 (χ^2^ = 13.3; *p* < .001). Instead, as would be expected by chance, a similar number of positive and negative correlations are seen for orthographic language (positive: 22/40; χ^2^ = 0.2; *p* = .65), spoken language (positive 17/30, χ^2^ = 0.3; *p* = .60), STM, and inhibitory control (positive 15/35; χ^2^ = 0.4; *p* = .55). It is to be noted that cognitive domains with stronger associations with metabolic control are not necessarily those that are more impaired. There are strong correlations between Phe measures and tasks tapping memory and learning even though there were no group impairments in this domain.

**Figure 3 fig3:**
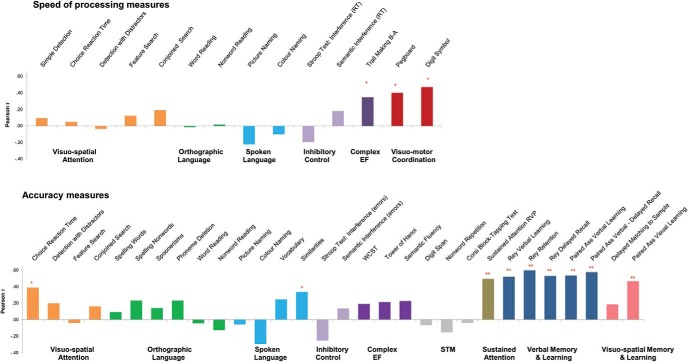
Correlations between current Phe and cognitive performance assessed by speed and accuracy measures. STM = short-term memory; EF = executive functions. See the online article for the color version of this figure.

We have also examined if effects of metabolic control were particularly evident for measures of speed of processing. Consistent with results from other studies, our adult PKU group was systematically impaired in speed of processing across domains (see Palermo et al., 2017). Comparing speed and accuracy measures is challenging. Within tasks, there may be speed–accuracy trade-offs with accuracy being at ceiling; between tasks, comparisons are more difficult. Still, if Phe levels mainly influenced speed of processing, differences between speed and accuracy measures should be observed across tasks. Figure 3 shows, as an example, correlation between speed and accuracy measures and levels of current Phe; patterns with other Phe measures are similar. It is clear, that, if anything, correlations are stronger with accuracy measures. In fact, consistent with other adult studies (Brumm et al., 2004; Channon et al., 2005), our results show no significant associations between speed in visuo-attentional tasks and current Phe levels. Significant correlations are seen in tasks tapping visuomotor coordination and the Trail Making Test B-A (see for similar results Moyle, Fox, Arthur, et al., 2007). These correlations, however, may be due to the fact that these tasks tap visuomotor coordination and complex executive functions, respectively, as much as to difficulties with speed of processing.[Fig-anchor fig3]

#### Differences between ages

Table 5 and Figure 2 show that associations between cognitive performance and metabolic control are present with measures taken at all ages, indicating that the history of metabolic control continues to influence performance even many years after measures were taken. There are, however, differences between different tasks and domains. Associations with Phe levels diminish with age in the case of visuospatial attention. Especially when performance is measured in terms of speed, correlations with current Phe are very limited. Similar results have been reported before (Channon et al., 2005; Moyle, Fox, Bynevelt, et al., 2007). The opposite is true in the case of tasks tapping memory and learning, visuomotor coordination and sustained attention. Here, correlations with current Phe are generally very high and higher than with measures taken at previous times. Correlations in other domains do not show particular trends.

We have statistically analyzed the relative contribution of metabolic control at different ages with a series of forward linear regression analyses with cognitive performance as the dependent measure and metabolic measures at different ages as predictors. Different regressions have been carried out with Phe averages and Phe fluctuations. Results are reported in Table 6. Visuospatial attention is best predicted by childhood measures (both Phe average and variation). Visuomotor coordination, sustained attention, and verbal memory and learning are best predicted by current Phe levels, but also by Phe fluctuations, respectively, during lifetime, adulthood, and childhood. Executive functions are best predicted by adolescent Phe average as well as by childhood Phe fluctuation. FSIQ is best predicted by life span fluctuations.[Table-anchor tbl6]

Another way to understand whether adult Phe levels independently contribute to performance is to assess correlations after the contribution of Phe levels at an earlier age has been statistically partialed out. Therefore, we have also carried out correlations between cognitive measures and adult and current Phe averages/fluctuations after partialing out corresponding measures in adolescence. A number of correlations remained significant. Most of them involved memory and learning, sustained attention, and the digit symbol, a complex task that taps, among other skills, visuomotor coordination:

1 Phe level 17-to present after partialing out Phe level in adolescence: Rey Retention: *r* = .43, *p* = .02; Digit symbol: *r* = .43, *p* = .02;
2 Phe current after partialing out Phe level in adolescence: Rey Learning: *r* = .43, *p* = .02; Rey Retention, *r* = .58, *p* < .01; Rey Delayed Recall, *r* = .51, *p* < .01; Paired associates verbal learning, *r* = .45, *p* = .02; paired associates verbal delayed, *r* = .50, *p* < .01; sustained attention, *r* = .45, *p* = .01; choice reaction time-accuracy, *r* = .40, *p* = .02; digit symbol, *r* = .41, *p* = .03;
3 Phe *SD* 17-to present after partialing out Phe *SD in* adolescence: visual search simple RT, *r* = .38, *p* < .05; paired associates visual learning: *r* = .38, *p* = .04.

## General Discussion

We have carried out our investigation using a much larger set of tasks than most studies. This has allowed us to obtain three main findings. First of all, we found strong and widespread correlations between cognitive performance in adulthood and metabolic control during the life span, even in a group of relatively well controlled AwPKU. Metabolic control had an impact both when measured in terms of average Phe levels and Phe fluctuations. A few studies have found an impact of Phe fluctuations on IQ and executive functions in children (for IQ, see Anastasoaie, Kurzius, Forbes, & Waisbren, 2008; Burgard et al., 1996; Hood et al., 2014; Vilaseca et al., 2010, for a trend approaching significance; for executive functions, see Arnold et al., 1998; Hood et al., 2014; but see and Viau et al., 2011, for negative results). We have demonstrated that fluctuations at different times during the life span have an impact on several cognitive measures even in adulthood, reinforcing the importance of this parameter.

Second, the tasks returning stronger correlations with metabolic measures were not those showing more significant group impairments. Phe levels are elevated even in individuals with the best dietary control. It is possible that certain functions (like speed of processing) show a generalized impairment due to elevated Phe levels in childhood and across the life span. Instead, it is functions where the majority of individuals perform well and only a few are impaired which allow the variability necessary for significant correlations to emerge. This is important when deciding how to monitor cognitive health and efficacy of treatment.

Finally, we found that the effect of metabolic control on adult cognition varied depending on the age when metabolic control was measured and on the particular cognitive domain examined. Tasks tapping verbal memory and learning, visuomotor coordination, and sustained attention were predicted more by recent metabolic control than by childhood control, so that current Phe level was the best predictor of performance. Instead, tasks tapping visuospatial processing and complex executive functions showed stable or decreasing associations with metabolic control across ages. There were no correlations with measures of orthographic processing. This was important. We are interested in a causal relationship between metabolic control and cognitive performance. However, both of these measures may be causally related to a third one: socioeconomic status, with no direct relationship. Better cognition and better metabolic control could co-occur in individuals with higher socioeconomic status. However, this hypothesis predicts that Phe levels will be strongly associated with orthographic processing which is a good reflection of socioeconomic status (e.g., see Hartas, 2011). The fact that this was not the case reassures us that correlations truly indicate the influence of metabolic control on cognition.

The impact of current Phe levels on sustained attention has been noted before (ten Hoedt et al., 2011; Schmidt et al., 1994), but an impact on memory and learning has not. In fact, some studies have reported no significant correlation (Brumm et al., 2004; Smith et al., 1996; but see Moyle, Fox, Bynevelt, et al., 2007). However, as we have noted, correlations depend on variability, and no correlation may result when Phe levels are high enough so that performance is systematically impaired across a group. It is interesting to note that in the studies where no correlation was reported (Brumm et al., 2004; Smith et al., 1996), there were significant group impairments and the current average Phe level was higher than in our sample (average Phe 1038; and 1554 μmol/L). Instead, in the study by Moyle, Fox, Bynevelt, et al. (2007) where, like ours, there was a significant correlation, there was no group impairment (unfortunately current Phe level was not reported).

There are some plausible reasons why different tasks show associations with Phe at different ages. The limited correlation between adult Phe levels and visuo-spatial speed measures is striking given the marked and pervasive impairments in our participants. The same result, however, has been reported by other studies: there is a reduced speed of processing in AwPKU but no correlations with adult Phe levels (Channon et al., 2005; Moyle, Fox, Bynevelt, et al., 2007). It is possible that speed deficits are caused by structural myelin damage which occurs early in life and/or is consolidated across many years of suboptimal Phe levels, making it difficult to modulate it through control in later years (see, Anderson & Leuzzi, 2010). Other functions, such as memory and learning, visuomotor coordination, and sustained attention, could be more sensitive to adult Phe because of more plastic effects on dopamine levels. PKU affects dopamine in two ways (see Lykkelund et al., 1988; Puglisi-Allegra et al., 2000; Landvogt et al., 2008): first because Phe cannot be converted into tyrosine which is a precursor of dopamine; and second because both Phe and tyrosine compete to pass the blood–brain barrier, leading to high levels of Phe further reducing the availability of tyrosine (see McKean, 1972; Burlina et al., 2000). Neurons in prefrontal cortex have a faster firing rate and more rapid dopamine turnover (Bannon et al., 1981; Tam et al., 1997). Thus, even small decreases in dopamine could impact functions supported by prefrontal areas, such as sustained attention and visuomotor coordination (for a particular relation between dopamine and frontal functions, see Diamond & Baddeley, 1996; Floresco & Magyar, 2006). The importance of dopamine levels for sustained attention and visuomotor coordination is well established (e.g., Nieoullon, 2002), but recent studies have also implicated the dopaminergic system in memory and learning (Ewell & Leutgeb, 2014; McNamara, Tejero-Cantero, Trouche, Campo-Urriza, & Dupret, 2014).

The impact of metabolic control may also depend on when brain areas are myelinated. Posterior regions supporting visuospatial processing will be myelinated first and, thus, an effect of Phe should be more evident in childhood. Frontal regions are myelinated later and, thus, an effect on executive functions should be seen in adolescence and adulthood (Best & Miller, 2010; Gogtay et al., 2004; Klingberg et al., 1999). This contrast accounts well for our results, which show that childhood Phe levels are most important for visuospatial attention whereas adolescence Phe levels are most important for complex executive functions. Clearly, however, myelin-based and dopamine-based mechanisms of damage do not need to be mutually exclusive. Although main myelination occurs at different times for different brain areas, recent studies have shown that it does not terminate in adolescence. Far from it, myelin continues to become thicker and more complete after learning throughout the life span to enable strengthening of relevant connections (see Wang & Young, 2014; Zatorre, Fields, & Johansen-Berg, 2012). Thus, high levels of Phe can impact on the brain in a variety of ways affecting both myelinization and levels of dopamine, resulting in different effects depending on type of task and developmental stage.

In conclusion, our results have both clinical implications for the management of individuals with PKU and theoretical implications for our understanding of this disease. The literature so far has provided inconsistent evidence on the type and extent of cognitive impairments in AwPKU and on the relation with Phe blood concentrations. This has resulted in a lack of agreement on how AwPKU should manage their diet (Hanley, 2004; Hoeks, den Heijer, & Janssen, 2009; Trefz, Maillot, Motzfeldt, & Schwarz, 2011). Although some clinicians advocate strict adherence to diet (e.g., ten Hoedt et al., 2011), others have suggested a more permissive and flexible approach (e.g., Channon et al., 2007). Our results are far from conclusive, but add to previous studies in suggesting that it is important to maintain low blood Phe through life, not just in childhood, as well as to maintain stable levels and minimize Phe fluctuations (for a similar conclusion, see also the meta-analysis of IQ by Lindegren et al., 2012). Our results also invite caution when selecting tasks to monitor performance. Tasks where there are more significant and consistent group impairments may not be the more sensitive to treatment outcomes. The same caution may apply to other clinical populations. Finally, our results highlight how the relation between metabolic control and cognitive functioning is complex and may depend on an interaction between cognitive domain and developmental stage. They show not only how effects of Phe change throughout the life span, but also how these effects may be specific for different cognitive domains.

Our study has a number of limitations (including relative arbitrariness of the cognitive domains, limited number of participants and the possibility of false positives given the large number of comparisons carried out). Further studies, should confirm our results. Our study, however, shows how careful analyses of impairments seen in inherited metabolic disorders like PKU may shed precious light on how healthy cognition is developed and maintained by the brain. Studies that investigate the effect of biochemical damage on brain functioning should complement more traditional studies looking at damage to structural brain areas. This will allow us to reach a more complete and integrated understanding of the neurophysiological basis of cognitive functioning.

## Figures and Tables

**Table 1 tbl1:** Demographic Information and Metabolic Control in Phenylketonuria Participants

Variable	*M*	*SD*	Range
Age	27.5	*7.3*	18–41
Education years	14.4	*1.9*	11–18
Gender (M/F)	13//24		
Childhood (Mean *N* obs. = 197; *SD* = 165)			
Phe average	432	*243*	180–1298
Phe variation	205	*63*	97–393
Adolescence (Mean *N* obs. = 77; *SD* = 70)			
Phe average	721	*340*	125–1420
Phe variation	157	*58*	28–284
Adulthood (Mean *N* obs. = 65; *SD* = 74)			
Phe average	802	*324*	188–1465
Phe variation	137	*68*	22–433
Lifetime (Mean *N* obs. = 340; *SD* = 241)			
Phe average	634	*291*	200–1188
Phe variation	164	*58*	57–324
Current Phe	720	*343*	65–1465
*Note.* Childhood = 0–10 years old; adolescence: 11–16 years old; Adulthood = 17+; *N* obs. = average *N* of measures for each participant in each time band; Phe average = average of median of yearly values; Phe variation = *SD* of yearly values; Current Phe = Phe at time of testing. Blood Phe measured in μmol/L.			

**Table 2 tbl2:** Correlations Between Phe Blood Concentrations (Phe Average and Phe Variation) at Different Ages

Variable	1	2	3	4	5	6	7	8	9
Phe average									
1. 1–10 years	—								
2. 11–16 years	.65**	—							
3. 17+	.47**	.79**	—						
4. Lifetime	.76**	.81**	.77**	—					
5. Current	.23	.55**	.76**	.48**	—				
Phe variation									
6. 1–10 years	.66**	.66**	.58**	.68**	.42*	—			
7. 11–16 years	.07	.28	.37*	.08	.28	.73**	—		
8. 17+	.14	.41*	.32	.28	.26	.49**	.61**	—	
9. Lifetime	.21	.44*	.43**	.19	.34*	.92**	.90**	.72**	—
* *p* < .05 (2-tails). ** *p* < .01 (2-tails).									

**Table 3 tbl3:** Full Scale Intelligence Quotient and Average Z Scores on Each Cognitive Domain for Adults With PKU Compared to Controls

Cognitive domain	*n*	*M*	*SD*	*p* difference with controls
Full Scale IQ	37	103.9	14.3	.003
Visuo-spatial attention	37	.5	.7	.005
Visuo motor coordination	37	1.1	1.6	.001
Complex EF	37	.8	1.2	.002
Inhibitory control	31	.2	.7	.12
Short-term memory	37	.6	.9	.005
Sustained attention	37	.6	1.3	.03
Orthographic processing	35	.5	1.2	.04
Spoken language	37	.7	1.0	.001
Verbal memory and learning	37	.0	.9	.88
Visuo-spatial memory and learn.	31	.5	1.3	.06
Overall z scores	37	.5	.7	<.001
% of impaired scores (Z score >2)	37	13.2	15.1	.001
*Note.* Results are presented as phenylketonuria (PKU Z) scores from the control group. To facilitate interpretation, for all scores, higher Z-score reflect worse performance. Visuo-spatial attention = Reaction time (RT) and accuracy in Simple Detection, Choice Reaction Time, Detection with Distractors, Feature Search, Conjoined Search. Visuo-motor coordination = Grooved Pegboard Test, Digit symbol. Complex executive functions (EF) = The Wisconsin Card Sorting Test-64 Card Version score, Tower of Hanoi scores; Semantic fluency, Trail Making B-A. Inhibitory controls = Stroop interference effect; Semantic interference effects (both RTs and accuracy). Short-term memory = Digit Span, Nonword repetition, Corsi Span. Sustained attention = Rapid Visual Information Processing. Orthographic language = RT and accuracy in words and nonword reading, accuracy in word and nonword spelling, phoneme deletions, and spoonerisms. Spoken language = RT and accuracy in picture naming and color naming, Vocabulary Wechsler Abbreviated Scale of Intelligence (WASI), and Similarities WASI. Verbal memory and learning = The Rey Auditory Verbal Learning Test and Paired Associates Verbal Learning. 10. Visuo-spatial memory and learning = Delayed Matching to Sample and Paired Associates Visual Learning.				

**Table 4 tbl4:** Demographic Information, Metabolic Control, and General Cognitive Performance for Adult Phenylketonuria Participants With Good vs. Poor Phe Control (<650 vs. >950 μmol/L Phe Average)

Variable	Lower-Phe (*n* = 14)		Higher-Phe (*n* = 14)		Higher vs. lower *t* test and *p* value
	*M*	*SD*	*M*	*SD*	
Age	23.7	6.8	30.5	6.9	*t*_(1.26)_ = −2.6; *p* = .01
Education	14.2	1.8	13.7	1.7	*t*_(1.25)_ = .2; *p* = .45
Gender (M/F)	5/9		5/9		
Childhood					
Phe average	323	116	494	219	*t*_(1.22)_ = −2.5; *p* = .02
Phe variation	173	46	246	65	*t*_(1.22)_ = −3.2; *p* = .004
Mean *N* obs. per participant	284	149	152	155	*t*_(1.26)_ = 2.3; *p* = .03
Adolescence					
Phe average	432	234	964	269	*t*_(1.23)_ = −5.3; *p* < .001
Phe variation	142	43	195	51	*t*_(1.23)_ = −2.8; *p* = .01
Mean *N* obs. per participant	106	78	73	66	*t*_(1.26)_ = 1.2; *p* = .24
Adulthood					
Phe average	461	135	1140	135	*t*_(1.26)_ = −13.3; *p* < .001
Phe variation	127	40	165	93	*t*_(1.26)_ = −1.4; *p* = .16
Mean *N* obs. per participant	73	72	67	89	*t*_(1.26)_ = .2; *p* = .85
Lifetime					
Phe average	536	182	823	162	*t*_(1.26)_ = −4.4; *p* < .001
Phe variation	145	41	197	59	*t*_(1.26)_ = −2.7; *p* = .01
Mean *N* obs. per participant	463	244	292	222	*t*_(1.26)_ = 1.9; *p* = .06
Current Phe	457	271	957	294	*t*_(1.24)_ = −4.5; *p* < .001
Full Scale IQ	106.9	10.6	96.4	16.6	*t*_(1.26)_ = 2.0; *p* = .06
Overall z score	.3	.3	1.0	.9	*t*_(1.26)_ = −2.8; *p* = .009
% of impaired scores (Z score > 2)	7.3	5.6	22.5	20.3	*t*_(1.26)_ = −2.7; *p* = .01
*Note.* Childhood = 0–10 years old; adolescence: 11–16 years old; Adulthood = 17+; *N* obs. = average *N* of measures for each participant in each time band; Phe average = average of median of yearly values; Phe variation = *SD* of yearly values; Current Phe = Phe at time of testing.					

**Table 5 tbl5:** Correlations Between Performance in Different Cognitive Domains and Measures of Metabolic Control

Variable	FSIQ	Visual attention	Visuo-motor coord.	Complex EF	Inhibitory control	STM	Sustained attention	Orthogr. language	Spoken language	Verbal M&L	Visuo-spatial M&L
Phe average											
Childhood	.12	.40*	.19	.21	.10	−.03	.01	−.09	.20	.08	.34
Adolescence	.28	.29	.34*	.40*	−.04	.03	.24	−.04	.26	.35	.56**
Adulthood	.33*	.38*	.39*	.26	−.05	.05	.33*	.21	.28	.47**	.48**
Lifetime	.20	.39*	.35*	.27	.22	.02	.18	.14	.27	.30	.40*
Current	.36*	.29	.47**	.30	.05	−.14	.49**	.08	.28	.67**	.39*
Phe variation											
Childhood	.49**	.37*	.49**	.49**	.14	.27	.15	.03	.43*	.46*	.60**
Adolescence	.56**	.23	.49**	.42*	.33	.27	.29	.22	.51**	.41*	.43*
Adulthood	.42**	.16	.46**	.32	.20	.22	.40*	.06	.53**	.03	.36*
Lifetime	.59**	.24	.50**	.41*	.13	.27	.33*	.13	.48**	.45*	.52**
Phe average + variation											
Childhood	.36*	.23	.22	.29	.22	.14	.07	.04	.29	.24	.39*
Adolescence	.48**	.33	.48**	.50**	.11	.14	.34	.10	.43*	.48**	.62**
Adulthood	.47**	.33*	.52**	.35*	.07	.17	.45**	.20	.50**	.37*	.52**
Lifetime	.51**	.40*	.55**	.44**	.23	.19	.33*	.16	.48**	.49**	.59**
*Note.* Childhood = 0–10 years old; Adolescence = 11–16 years old; Adulthood = 17+; Phe average = average of median of yearly values; Phe variation = Average *SD* of yearly values; Current Phe = Phe at time of testing; FSIQ = Full Scale IQ; EF = executive functions; STM = short-term memory. For the tasks included in each cognitive domain see Table 2 footnote. The number of phenylketonuria participants is slightly different across tests (see Table 2) and as well as the number of Phe measures available (see Table 1), this means that the same value of r may have different probabilities. *N* of correlations = 143; *N* of significant correlations: 66/143 = 43%; lifetime: 8 /11 = 73%; current 5/11 = 46%.											
* *p* < .05. ** *p* < .01.											

**Table 6 tbl6:** Linear Regression Analysis Forward Method

Cognitive domain	Phe average				Phe variation			
	Full-model	Significant variable/s	*R*^2^	*B*	Full- model	Significant variable/s	*R*^2^	*B*
Full Scale IQ	*ns*				.001	Lifetime Phe	.34	.14
Visuo-spatial attention	.03	Childhood Phe	.16	.1	.04	Childhood Phe	.14	.4
Visuo motor coordination	.01	Current Phe	.22	.2	.005	Lifetime Phe	.25	1.3
Complex executive functions	.03	Adolescence Phe	.16	.1	.006	Childhood Phe	.24	.9
Inhibitory control	*ns*				*ns*			
Short-term memory	*ns*				*ns*			
Sustained attention	.006	Current Phe	.24	.2	.03	Adult Phe	.16	.8
Orthographic language	*ns*				*ns*			
Spoken language	*ns*				.003	Adolescence Phe	.28	.8
Verbal memory and learning	<.001	Current Phe	.44	.2	.02	Childhood Phe	.21	.6
Visuo-spatial memory and learning	.002	Adolescence Phe	.32	.2	<.001	Childhood Phe	.36	1.2
*Note*. Performance in different cognitive domains predicted by Phe average or Phe variation for different developmental epochs: early childhood (up to 10 years) adolescence (11–16) and adulthood (17+); measures of current Phe and life span Phe measures are also included. *B* shows changes in performance measures for every 100 μmol/l of Phe (e.g., for visuo-motor coordination, .2 z scores are added for every 100 μmol/l of phe; for IQ, 14 point of IQ are lost for every 100 μmol/l of Phe.
